# ATPR‐induced G_0_/G_1_ phase arrest in gastric cancer cells by regulating the binding of 14‐3‐3ε and filamin A

**DOI:** 10.1002/cam4.1583

**Published:** 2018-06-04

**Authors:** Yingli Zhao, Xing Fang, Hui Fang, Yubin Feng, Feihu Chen, Quan Xia

**Affiliations:** ^1^ Department of Pharmacy The First Affiliated Hospital of Anhui Medical University Hefei China; ^2^ College of Pharmacy Anhui Medical University Hefei China

**Keywords:** 14‐3‐3ε, ATPR, filamin A, gastric cancer, targeted proteomics

## Abstract

4‐amino‐2‐trifluoromethyl‐phenyl retinate (ATPR) was able to induce the G_0_/G_1_ phase arrest in gastric cancer SGC‐7901 cells by downregulating 14‐3‐3ε. However, the mechanisms underlying this effect have not been fully elucidated. Because 14‐3‐3ε functions as a molecular chaperone on cell cycle regulation, the interaction between 14‐3‐3ε and the target proteins is worth an in‐depth study. In this study, the use of targeting proteomics identified 352 14‐3‐3ε‐binding proteins in SGC‐7901 cells. Analysis of gene ontology (GO) was performed using PANTHER to annotate the biological processes, protein classes, and pathways of these proteins. In 25 cell cycle‐related proteins, filamin A was reduced following ATPR treatment, and this change was validated by immunoprecipitation. The cell cycle was arrested at the G_0_/G_1_ phase following ATPR treatment or filamin A silencing in SGC‐7901 cells. Furthermore, subcellular expression analysis showed that 14‐3‐3ε and filamin A were transferred from the cytoplasm to the nucleus after ATPR treatment. On the other hand, overexpression of 14‐3‐3ε, in SGC‐7901 cells, resulted in an increase in the total cellular level of filamin A and an increase in the subcellular localization of filamin A in the cytoplasm. ATPR treatment of the 14‐3‐3ε overexpression cells decreased the total level of filamin A and redistributed filamin A protein from the cytoplasm to the nucleus. Immunohistochemical analysis showed that the expression levels of 14‐3‐3ε and filamin A in gastric cancer tissues were significantly higher, with a predominant localization in the cytoplasm, compared to the levels in matched tissues. Taken together, our results suggest that ATPR can induce nuclear localization of filamin A by reducing the binding of 14‐3‐3ε and filamin A, which may be the mechanism of ATPR‐induced G_0_/G_1_ phase arrest.

## 1 INTRODUCTION

Gastric cancer is the fifth most common cancer in the world and has received global concern as a health problem. According to the World Cancer Report in 2014, there were approximately 952 000 new cases of gastric cancer diagnosed worldwide in 2012, and the mortality was ranked third, immediately following lung cancer and liver cancer.^1^ For gastric cancer, early diagnosis and surgical resection are currently considered the preferred treatment, and the 5‐year survival rate is more than 95%. However, due to the low rate of early diagnosis, most patients have advanced‐stage disease at diagnosis, and the main treatment is the combination of chemotherapy, molecular‐targeted therapy, and immunotherapy.^2^ Molecular‐targeted therapy provides an antitumor effect by regulating some key proteins in the cell cycle, cell growth, and apoptosis signaling pathways. Most notably, the emergence of trastuzumab initiated the molecular‐targeted therapy era. The initiation of G_1_ phase arrest and the induction of P27 are among its mechanisms.^3^ After decades of research on the physiological functions of cell cycle proteins and their relevance to cancer, targeting cell cycle‐dependent kinase (CDKs) inhibition has provided an attractive option in the treatment of cancer.^4^

Cell cycle regulation is complex, and various factors participate in the process, in which molecular chaperones play an important role. As molecular chaperones were discovered, aberrant expression of molecular chaperones has been widely reported in cancer tissues and cells.^5^ The 14‐3‐3 proteins, which belong to the molecular chaperones group, are a family of highly conserved acidic 30 kD molecules that are involved in the regulation of multiple signaling pathways in cells. 14‐3‐3 proteins can control cell cycle arrest and recovery through phospho‐dependent targeted binding to various protein kinases. The interactions between 14‐3‐3 isoforms and the target proteins reveal an abundance of potential drug targets that could be used to therapeutically treat disease caused by aberrant cell proliferation such as cancer.^6^

All‐trans retinoic acid (ATRA), a retinoid, is the first‐line drug for the treatment of acute promyelocytic leukemia (APL). The main mechanism of ATRA is through its binding to the receptors, including the retinoic acid receptor (RAR) and retinoid X receptor (RXR), to regulate the expression of the target gene, as well as to induce G_0_/G_1_ phase arrest. With the widespread use in clinical trials, the side effects and adverse reactions of ATRA, such as retinoic acid syndrome, drug resistance, and a high recurrence rate, affected its application in the clinical setting. ATRA has achieved remarkable effects in treatment of hematological malignancies, but for solid tumors (such as gastric cancer), its effect is far from being satisfactory. Therefore, developing safer and more effective drugs has become an urgent need.^7^ Based on our primary pharmacodynamics screening, treatment with 4‐amino‐2‐trifluoromethyl‐phenyl retinate (ATPR) for 48‐72 hours could induce G_0_/G_1_ arrest and inhibit proliferation in a variety of tumor cells, including gastric cancer cells.^8^, ^9^, ^10^, ^11^ Our recent study showed that ATPR was able to induce the G0/G1 phase arrest in gastric cancer SGC‐7901 cells by downregulating 14‐3‐3ε. As a matter of fact, we did find the effect of ATPR on cell cycle was similar to ATRA, but they exhibited different mechanisms of action on cell cycle‐related proteins. ATPR could modulate the cell cycle‐related proteins of the PI3K‐AKT‐FOXO signaling pathway by downregulating 14‐3‐3ε expression. This effect resulted in increased FOXO1 and P27kip1 and in decreased CDK2 and cyclin E expression. However, the mechanism by which 14‐3‐3ε affects the cell cycle requires further study.^12^

In this study, targeted proteomics was used to find the 14‐3‐3ε‐binding proteins in gastric cancer SGC‐7901 cells, and this process identified 25 cell cycle‐related proteins. In addition, the combination of 14‐3‐3ε and filamin A, an actin cross‐linking protein identified as a CDK1‐binding partner, was reduced after ATPR treatment. Therefore, we found that ATPR might play a role in G_0_/G_1_ cycle arrest by reducing the binding of filamin A and 14‐3‐3ε and regulating the subcellular localization of filamin A. IHC analysis in this study shows that 14‐3‐3ε and filamin A proteins are both overexpressed in gastric cancer tissues compared to adjacent nontumor counterparts. Our results implied that downregulating the binding of 14‐3‐3ε to cell cycle‐related targeted proteins might have an antitumor effect.

## 2 MATERIALS AND METHODS

### 2.1 Cell culture, treatment, extraction of nuclear, and cytoplasmic proteins

Human gastric adenocarcinoma SGC‐7901 cells (the Shanghai Institute of Cell Biology, Chinese Academy of Sciences) were grown in DMEM containing penicillin and streptomycin (each 100 mg/L) and supplemented with 10% fetal bovine serum (FBS) at 37°C in 5% CO_2_. ATPR was synthesized by the School of Pharmacy, Anhui Medical University, with a purity of 99%. ATPR was prepared as a stock solution of 10^−2^ mol/L in dehydrated alcohol and kept at −20°C. Cells were treated with ATPR (10^−5^ mol/L final concentration, the ATPR group) for 48 hours and then collected. The vehicle group (0.1% alcohol diluted with DMEM) was set up, and the cells were treated under the same conditions. Cell nuclear and cytoplasmic proteins were extracted using nuclear and cytoplasmic extraction reagents (KeyGEN BioTECH, Nanjing, China).

### 2.2 Immunoprecipitation and Western blot

Total proteins were collected by centrifugation for 15 minutes at 14 000 *g* at 4°C. The supernatant was collected, and the protein concentrations were determined using a BCA Protein Assay Kit (Beyotime, Shanghai, China) with BSA as the standard.

For immunoprecipitation, the supernatants of all groups were first diluted to 2 mg/mL to take each of them to a volume of 2 mL for the next step. The anti‐14‐3‐3ε or antifilamin A antibody (Abcam, Cambridge, UK) was added to the diluted 2‐mL supernatant of the ATPR group. The 4‐mL supernatant of the vehicle group was divided into two equal parts, adding normal rabbit IgG (Abcam, Cambridge, UK) and anti‐14‐3‐3ε or antifilamin A antibody to each. The supernatant and antibody were incubated overnight at 4°C and then added to 80 μL Protein A/G‐plus agarose beads (Thermo Fisher Scientific). They were incubated 2 hours at 4°C. The beads were collected by centrifugation for 5 minutes at 200 *g* and washed three times with NP‐40. Finally, the supernatants were discarded. 2 × loading buffer was added to the beads with boiling water for 5 minutes. The obtained samples were subjected to vertical electrophoresis, and the gel was stained with Coomassie Brilliant Blue dye (TIANGEN, Beijing, China) or immunoblotted.

For Western Blot, equal amounts of protein were separated on 12% SDS‐PAGE and transferred to a 0.45 μm PVDF membrane (Millipore, USA) followed by blocking in 5% skim milk in TBST at room temperature. The membranes were incubated overnight at 4°C with anti‐14‐3‐3ε (1:1000, Abcam, Cambridge, UK), antifilamin A (1:100, Santa Cruze, USA), anti‐GAPDH (1:500, Elabscience, Wuhan, China), and anti‐H3 (1:1000, Abcam, Cambridge, UK). The membranes were washed in TBST and incubated with secondary antibody (1:10 000) for 1 hour at room temperature followed by exposure to electrochemiluminescence. Finally, ImageJ was used to measure the protein bands.

### 2.3 In‐gel enzymatic digestion and mass spectrometry analysis

The protein bands were excised from the one‐dimensional Coomassie blue‐stained polyacrylamide gel. The bands were digested in the gel with an excess of sequencing‐grade trypsin (Promega, USA).^13^ Each 4‐μg sample was loaded and separated on a C18 column (10 cm × 100 μm) using a nano‐liquid chromatograph (Dionex, Thermo Fisher). The separation procedure refers to our previous experimental conditions.^12^ The liquid phase‐separated peptide was introduced into a Q‐Exactive tandem mass spectrometer (ThermoFisher Scientific, San Jose, CA) equipped with an ESI ionization source.

### 2.4 Identification of proteins and bioinformatics analysis

The raw data files from samples and BSA were analyzed using the SEQUEST (v.1.13, ThermoFisher Science) search engine and the Proteome Discoverer (v.2.1, ThermoFisher Science) using the human nonredundant peptide database obtained from the UniProt human database (Nov 3, 2014, 88 717 sequences). The UniProt accessions of identified proteins were uploaded on PANTHER (Protein Analysis THrough Evolutionary Relationships, http://pantherdb.org) classification systems.

### 2.5 Double immunofluorescent staining

For double immunofluorescent staining, SGC‐7901 cells were seeded in a six‐well plate and fixed in 4% ice‐cold paraformaldehyde for 10 minutes after overnight culturing. Afterward, the cells were blocked with 10% BSA for 10 minutes and incubated with antibodies against 14‐3‐3ε and filamin A overnight at 4°C. Then, the cells were incubated with FITC‐conjugated goat anti‐rabbit secondary antibody (1:200, 2 mg/mL, Zhongshan Jingqiao, China) and CY3‐conjugated goat anti‐mouse secondary antibody (1:200, 2 mg/mL, Zhongshan Jingqiao, China). DAPI (2 mg/mL, Beyotime, Shanghai, China) was used to counterstain the nuclei, and cells were visualized with a laser scanning confocal microscope (Olympus, China).

### 2.6 Overexpression or knockdown of 14‐3‐3ε and siRNA transfection to filamin A in SGC‐7901

For overexpression of 14‐3‐3ε, SGC‐7901 cells were maintained in 1 mL of complete medium with 5 mg/mL Polybrene per well and were treated with 3 × 10^6^ TU/mL 14‐3‐3ε gene‐lentiviral particles overnight, and three wells were transduced with empty lentiviral particles as the control.^12^ For knockdown of 14‐3‐3ε or filamin A, all sequences of siRNAs are shown in Table 1. The siRNAs (GenePharma, China) and Lipofectamine 2000 reagent (Invitrogen, USA) were diluted in Opti‐MEMI Reduced Serum Medium (Gibco, USA) separately and incubated for 5 minutes at room temperature. Then, the complex of siRNA‐lipofectamine was added into cells and cultured for 6 hours, which were successively grown in DMEM containing 7% FBS for another 24 hours. ATPR or 0.1% alcohol was added to the final concentration at 10^−5^ mol/L, and cells were cultured for 48 hours.

**Table 1:** The siRNA sequences of 14‐3‐3ε and filamin A

siRNA	sequences(5′‐3′)
YWHAE‐homo‐279	CCUCCUAUCUGUUGCAUAUTT
	AUAUGCAACAGAUAGGAGGTT
YWHAE‐homo‐590	GAACAGCCUAGUGGCUUAUTT
	AUAAGCCACUAGGCUGUUCTT
YWHAE‐homo‐685	CCGUAUUCUACUACGAAAUTT
	AUUUCGUAGUAGAAUACGGTT
Filamin A‐homo‐1451	CCACCUACUUUGAGAUCUUTT
	AAGAUCUCAAAGUAGGUGGTT
Filamin A‐homo‐2435	GCACUUACAGCUGCUCCUATT
	UAGGAGCAGCUGUAAGUGCTT
Filamin A‐homo‐2776	GCUGGCAGCUACACCAUUATT
	UAAUGGUGUAGCUGCCAGCTT

YWHAE: the gene encoding 14‐3‐3ε protein.

### 2.7 Cell cycle analysis

Specific procedures refer to the cell cycle kit (Beyotime, Shanghai, China) instructions. Cells were harvested and washed with PBS twice and then fixed with 70% ethanol for 12 hours. The fixed cells (1 × 10^6^/mL) were spun down and resuspended in PBS. After incubation with ribonuclease and propidium iodide at 37°C for 30 minutes, cells were filtered through a nylon mesh (BD Bioscience, USA) before analysis on a flow cytometer (BD Bioscience, USA).

### 2.8 Patient enrollment and tissue collection

This study was approved by the Human Research Ethics Committee of the First Affiliated Hospital of Anhui Medical University (China). Twenty paired human gastric cancer samples (GC) and their adjacent nontumorous gastric tissues (NT) were obtained from surgical resection performed at our hospital between March 2017 and June 2017. Patients who underwent primary surgical resection had not received radiotherapy and chemotherapy before surgery. The resected specimens were fixed in 10% formalin for paraffin embedding. Pathological examination was performed on all of the GC and the NT samples.

### 2.9 Immunohistochemistry assay

The immunohistochemical procedure was performed according to the general‐purpose SP kit (ZSGB‐BIO, China) instructions. Sections were incubated with anti‐14‐3‐3ε or antifilamin A at 4°C overnight, and each section was incubated with secondary antibody at room temperature for 30 minutes. For negative controls, the primary antibody was substituted with PBS. Each experiment was repeated three times, and images were taken using a microscope (Haimen Changlong, China). Semiquantitative integration method was adopted with modifications from Magara et al^14^ to determine the immunohistochemical staining positivity. Briefly, a value of 0, 1, 2, 3, or 4 was assigned according to the proportion of positive cells to the total cells in the observed field: less than 5%; 5%‐25%; 26%‐50%; 51%‐75%; and more than 75%, respectively. These values were then multiplied by the staining intensity of 0 (no staining), 1 (weak staining, light yellow), 2 (moderate staining, yellow brown), or 3 (strong staining, brown) to obtain a score ranging from 0 to 12. A score equal to or <3 was considered low expression, and a score >3 was considered high expression. Each slide underwent 5 random counts using the high‐power field (400×), and the average was taken. The examiner was not blinded for the study, and informed consent was obtained from all localization patients.

### 2.10 Statistical analysis

Data presented in images are from one representative experiment. All statistical analyses were carried out using SPSS version 17 (SPSS Inc., Chicago, IL, USA) and GraphPad Prism (GraphPad Software). All data are shown as the mean ± standard deviation. Each experiment was performed at least in triplicate, and the measurements were performed in three independent experiments. To compare the two groups, Student’s *t* test and one‐way analysis of variance were used.

## 3 RESULTS

### 3.1 352 proteins were deduced to be bound with 14‐3‐3ε in SGC‐7901 cells

**Figure 1:**
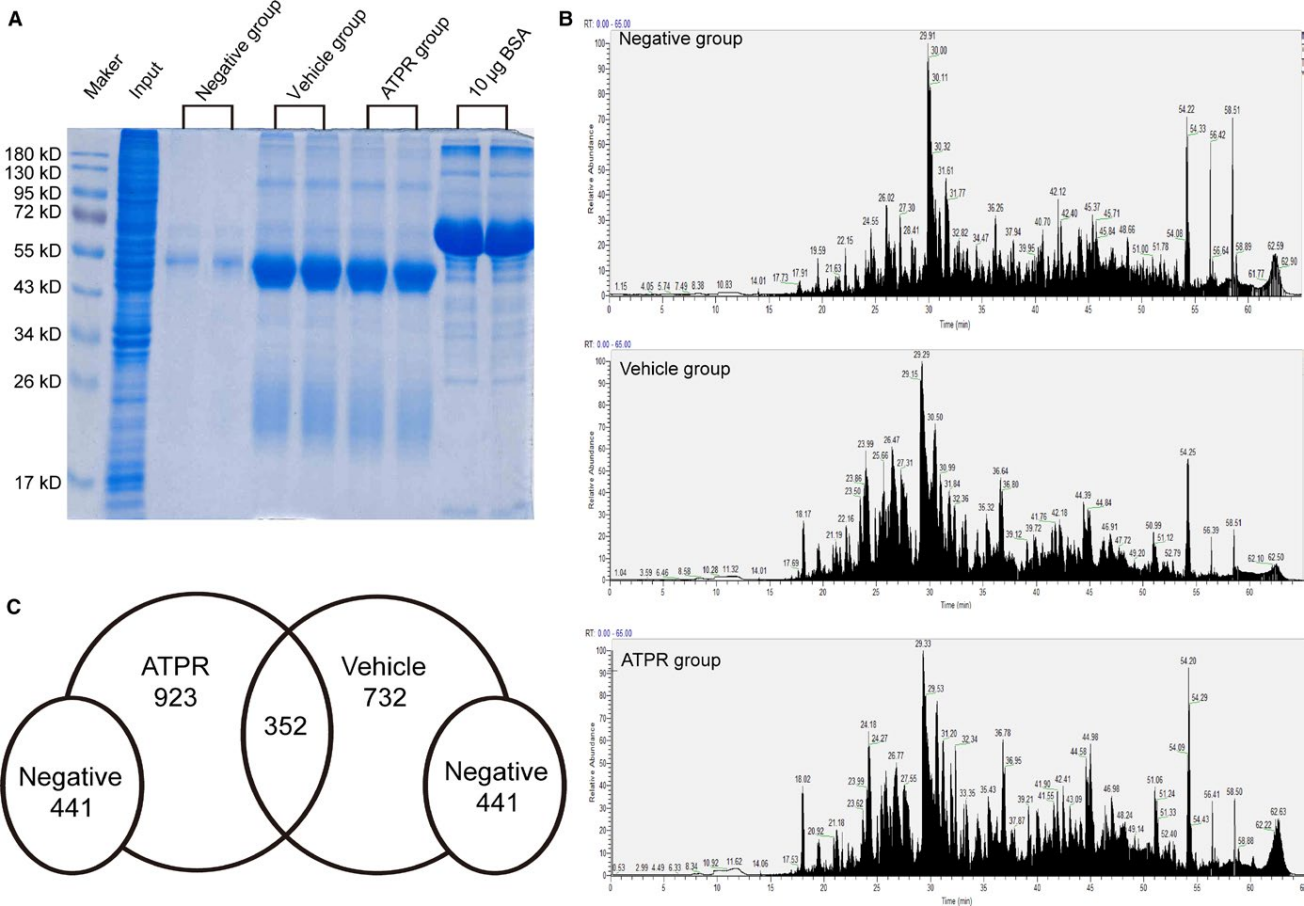
14‐3‐3ε‐binding proteins were identified using targeted proteomics. A, 12% SDS‐PAGE gel of immunoprecipitated productions with Coomassie blue‐stained. Lysates extracted from SGC‐7901 cells as positive control (Input), lysates immunoprecipitated with normal rabbit IgG (Negative group) or 14‐3‐3ε antibody (Vehicle group), and ATPR treatment followed by immunoprecipitation with 14‐3‐3ε antibody (ATPR group). BSA (10 μg) was used as a reference standard in following experiments. B, Bands were sliced and followed by in‐gel trypsin digestion, then were analyzed on a Q‐Exactive MS equipped with a Nano‐ESI to obtain TIC. C, A total of 1432 proteins and 1242 proteins were identified, respectively, in the ATPR group and the vehicle group at the confidence of FDR<1%, whereas 441 proteins were found in the negative group. Three hundred and fifty‐two proteins were deduced to be bound with 14‐3‐3ε

In our previous study, ATPR was shown to induce G_0_/G_1_ phase arrest in gastric cancer cells by downregulating the total expression of 14‐3‐3ε and inducing its nuclear location.^12^ To further explore the role of ATPR and 14‐3‐3ε on the cell cycle in gastric cancer, a targeted proteomics approach was used to identify the 14‐3‐3ε‐binding proteins in ATPR group and the vehicle group. We found that 1432 and 1242 proteins were identified in the ATPR group and the vehicle group, respectively, at the confidence of FDR<1%, whereas 441 proteins were found in the negative control group. By comparing these results, 352 proteins were deduced to be bound with 14‐3‐3ε (Figure 1C).

### 3.2 Cell cycle‐ and differentiation‐ related proteins were assigned by bioinformatics analysis

**Figure 2:**
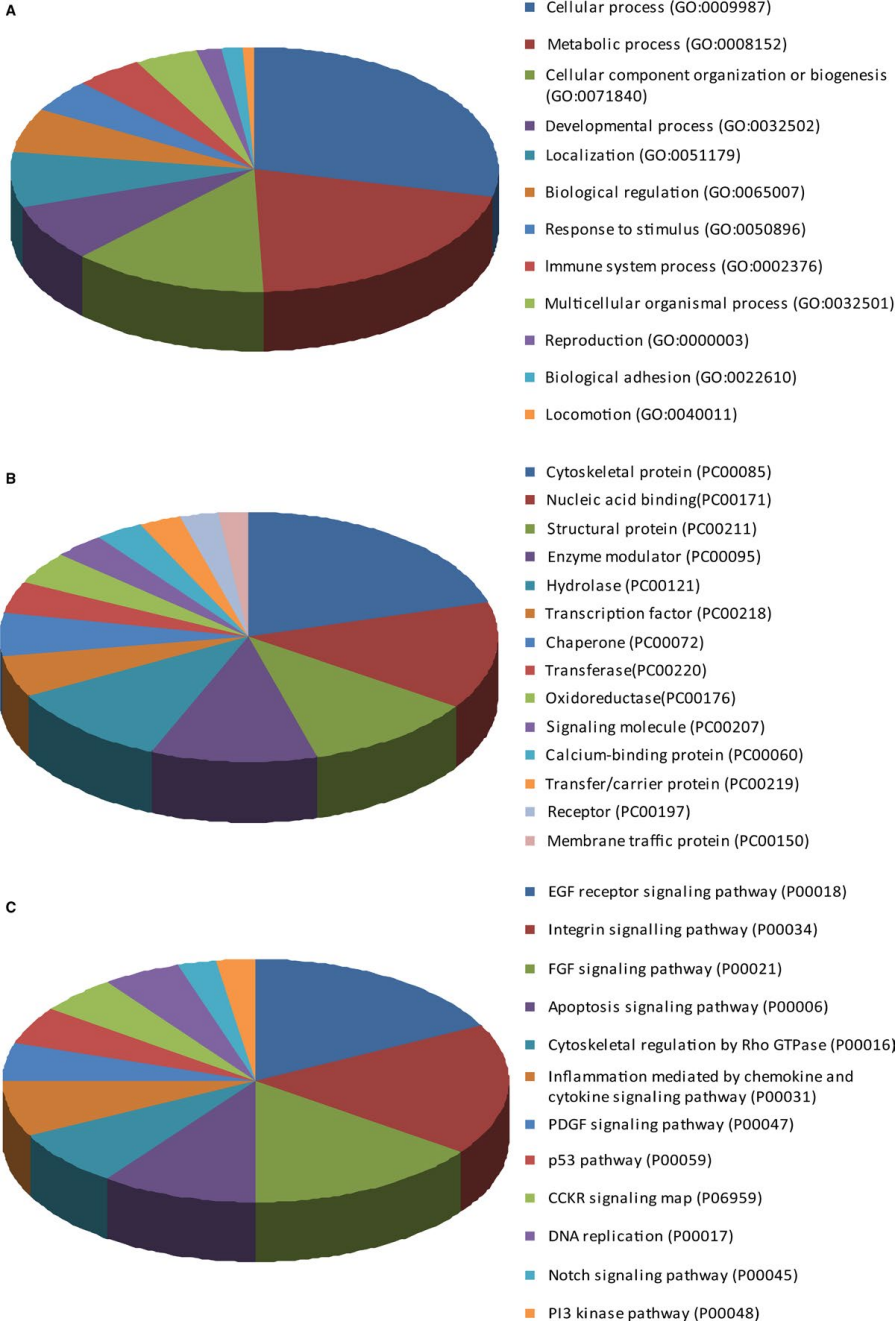
Bioinformatics analysis of the 14‐3‐3ε‐binding proteins. Gene ontology classification of identified proteins was annotated by PANTHER on the basis of biological processes (A), protein classes (B), and pathways (C)

To fully understand the function of the 14‐3‐3ε‐binding proteins founded in targeted proteomics, the 352 co‐expressed proteins were annotated with GO terms using PANTHER. The biological processes, protein classes, and pathways involved are shown in Figure 2. The biological processes obtained from GO analysis showed that these proteins were associated with the cellular process, the metabolic process, and the developmental process (Figure 2A). Notably, the majority of the differentially expressed proteins are cytoskeletal proteins, nucleic acid‐binding proteins, and enzyme modulator proteins (Figure 2B). For the pathway analysis, the result suggested that the proteins were involved in the EGF receptor signaling pathway, integrin signaling pathway, and FGF signaling pathway (Figure 2C). To further investigate the mechanism of ATPR‐induced cell cycle arrest, we must first focus on the relationships between the 14‐3‐3ε‐binding proteins and cell cycles. Therefore, the proteins involved in the cell cycle are listed in Table S1. In addition, in view of 14‐3‐3ε involving in ATPR‐induced differentiation of gastric cancer cells, we also listed those proteins which were involved in cell differentiation in Table S2. We found that some proteins played functions in both the cell cycle and in differentiation, such as filamin A, SFN, Catenin beta‐1, and ANXA1. This provided us the target proteins to further study.

### 3.3 The binding of 14‐3‐3ε and filamin A was validated by immunoprecipitation and double immunofluorescent staining

**Figure 3:**
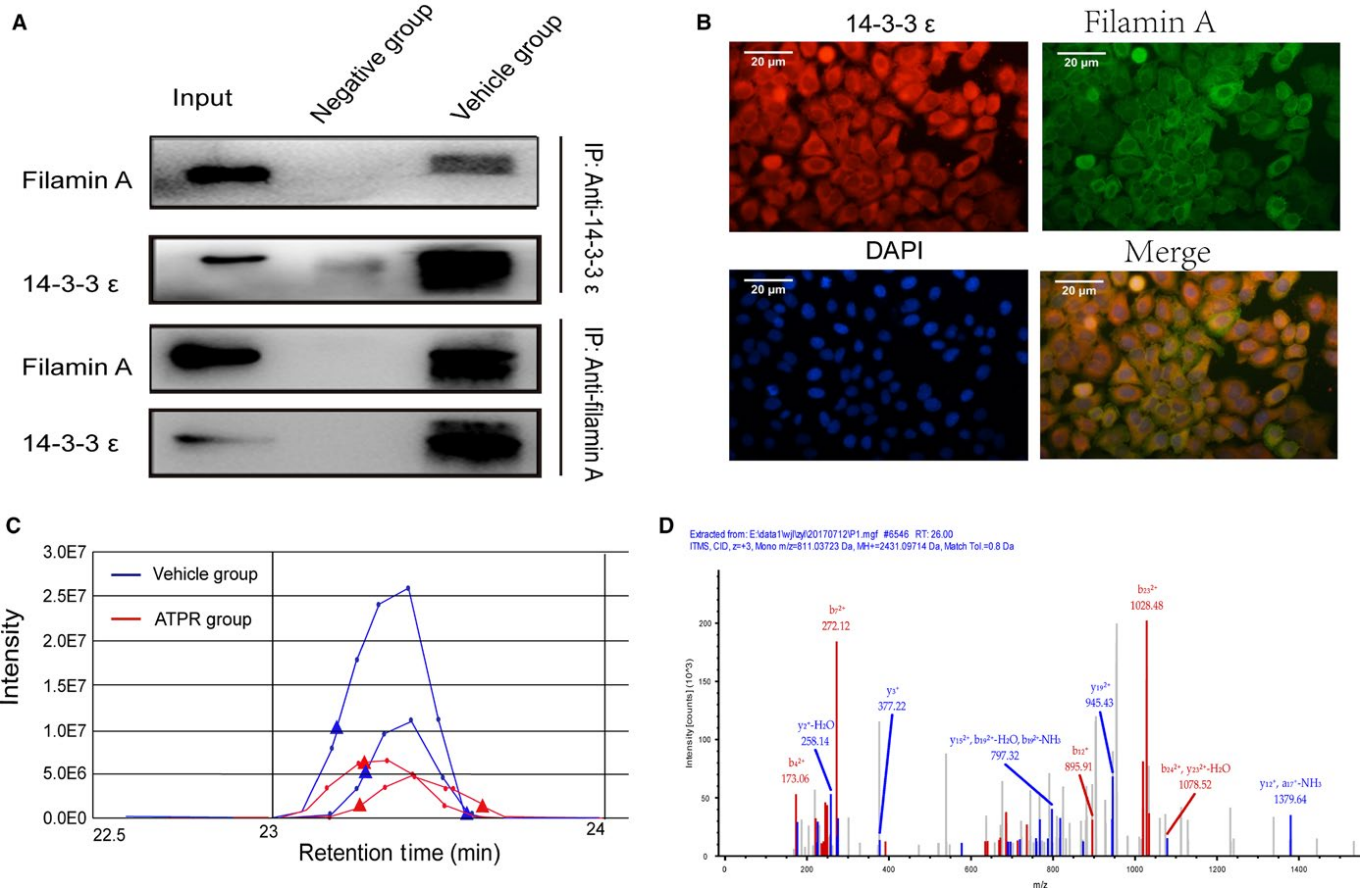
The binding of 14‐3‐3ε and filamin A was validated by immunoprecipitation and double immunofluorescent staining. A, The binding of filamin A and 14‐3‐3ε in immunoprecipitation complex was validated by Western blotting. B, Filamin A and 14‐3‐3ε colocalized in the cytoplasm. C, A label‐free quantification proteomics software SIEVE was used to analyze the differentially expressed filamin A between the vehicle group and the ATPR groups. Filamin A and 14‐3‐3ε were low‐expressed in the ATPR groups. D, Representative MS/MS spectra of peptide AGQSAAGAAPGGGVDTRDAEmPATEK from filamin A

In 14‐3‐3ε‐binding proteins, filamin A was chosen as the first to study. To verify the results of proteomics analysis, immunoprecipitation and immunofluorescence double staining were chosen to analyze the binding of filamin A to 14‐3‐3ε. Immunoprecipitation showed a significant binding of filamin A with 14‐3‐3ε (Figure 3A). At the same time, we used immunofluorescence double staining to track the distribution of filamin A and 14‐3‐3ε. The results showed that filamin A and 14‐3‐3ε colocalized in the cytoplasm, which further suggested the combination of filamin A and 14‐3‐3ε (Figure 3B). A significant reduction in the binding of filamin A and 14‐3‐3ε was also demonstrated when label‐free quantification proteomics software SIEVE was used to analyze the differentially expressed proteins between the vehicle group and the ATPR groups (Figure 3C). Representative MS/MS spectra of the peptide AGQSAAGAAPGGGVDTRDAEmPA TEK was from filamin A (Figure 3D). Taken together, all of these results confirmed the prior results of targeted proteomics and suggested that ATPR had an impact on this combination of filamin A and 14‐3‐3ε.

### 3.4 Filamin A was involved in ATPR‐induced SGC‐7901 cell cycle arrest

**Figure 4:**
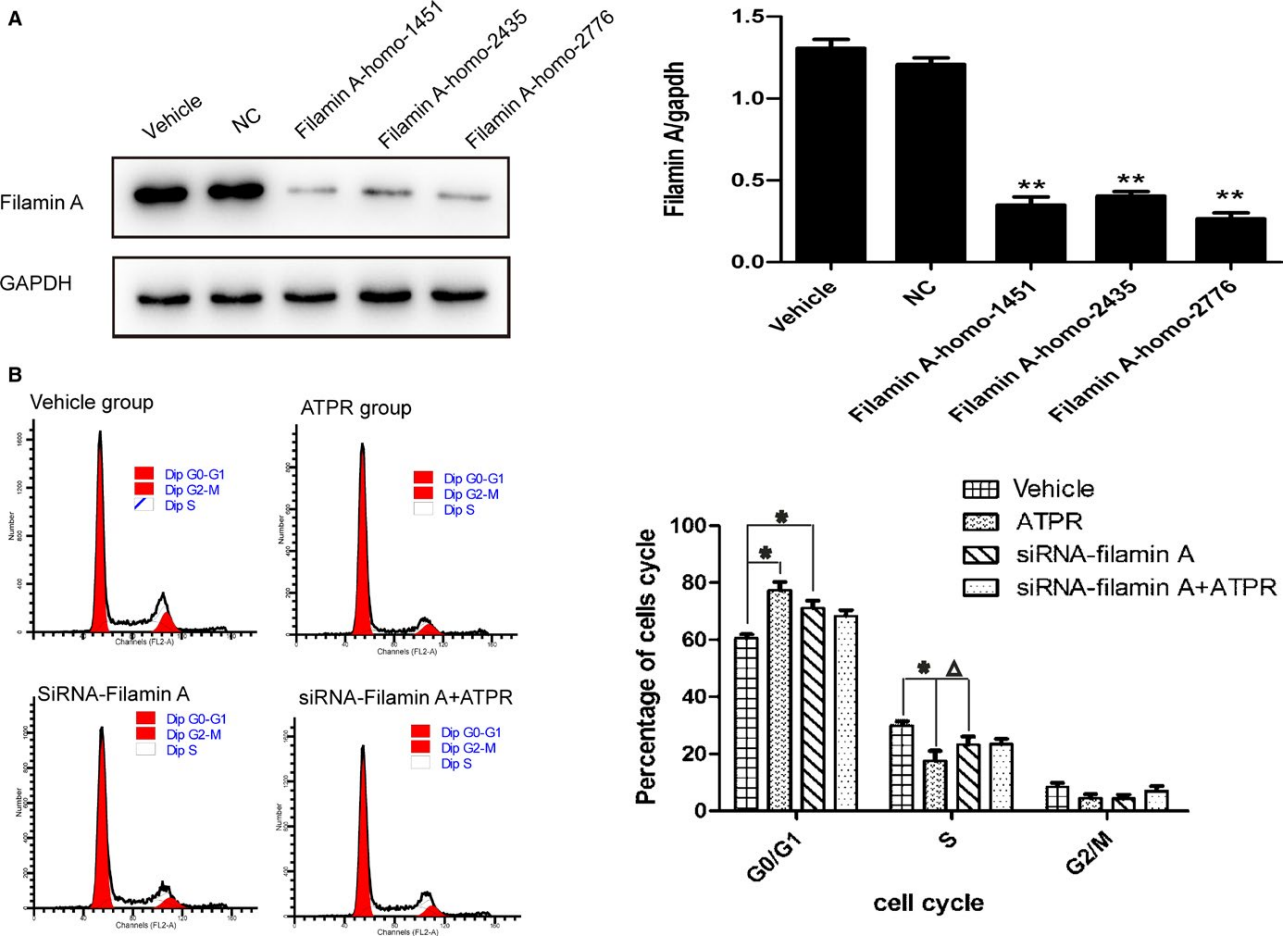
Filamin A was involved in ATPR‐induced G_0_/G_1_ phase arrest. A, Western blotting showed filamin A was successfully silenced with filamin A‐homo‐2776 sequence to the subsequent experiments. B, Cell cycle analysis in the vehicle group, the ATPR group, the siRNA‐filamin A group, and the siRNA‐filamin A+ ATPR group. Compared with the vehicle group, cell cycle was arrested at G_0_/G_1_ phase when ATPR‐induced or filamin A was silenced. Compared with the siRNA‐filamin A group, the G_0_/G_1_ and S phase distributions were not observably changed after ATPR treatment on siRNA‐filamin A cells. *P < .05, **P < .01, and ^△^ *P* > .05

To explore the role of filamin A in the cell cycle, we silenced the expression of filamin A in gastric cancer cell SGC‐7901 and then treated them with ATPR. As shown in Figure 4A, filamin A was decreased when silenced with three siRNA sequences, therefore the Filamin A‐homo‐2776 sequence was used for subsequent experiments. The cell cycle was arrested at the G_0_/G_1_ phase when it was ATPR‐induced or when filamin A was silenced (Figure 4B). Notably, compared with the siRNA‐filamin A group, the G_0_/G_1_ phase distribution was not observably changed after ATPR treatment on siRNA‐filamin A cells (Figure 4B). This indicated that filamin A had the effect of promoting cell cycle progression and played an important role in ATPR‐induced gastric cancer cell cycle arrest.

### 3.5 ATPR may regulate the subcellular localization of filamin A through 14‐3‐3ε

**Figure 5:**
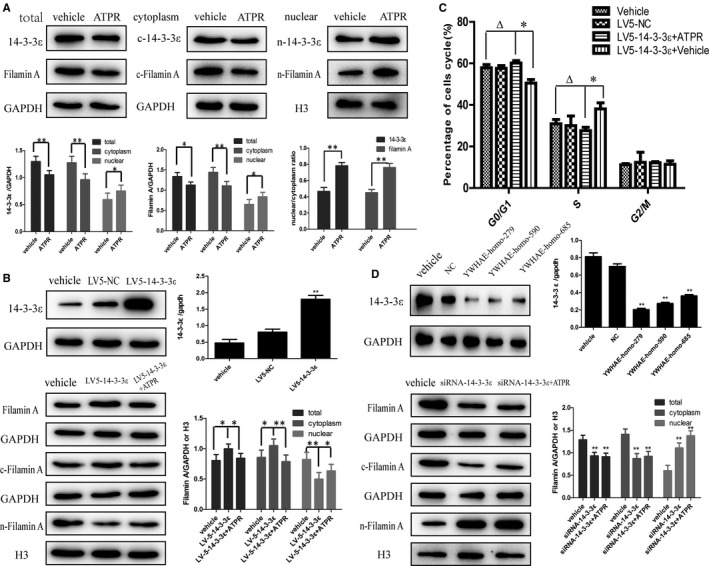
ATPR regulated the subcellular localization of filamin A through 14‐3‐3ε. A, Subcellular expression analysis of 14‐3‐3ε and filamin A after ATPR treatment. Compared with the vehicle group, ATPR can significantly decrease the total expression of 14‐3‐3ε and filamin A, decrease the expression of cytoplasmic 14‐3‐3ε and filamin A, and improve nuclear 14‐3‐3ε and filamin A (c‐: cytoplasm;n‐: nuclear). Compared with the vehicle group, it showed a significantly high nucleus to cytoplasm ratio for 14‐3‐3ε and filamin A in the ATPR group. B, The 14‐3‐3ε overexpression can significantly increase the total amount of cytoplasmic filamin A and decrease the expression of nuclear filamin A. This effect can be attenuated by ATPR treatment. C, The 14‐3‐3ε overexpression attenuated the effect of ATPR‐induced G_0_/G_1_ phase arrest. D, When 14‐3‐3ε was specifically silenced with siRNA transfection, the total amount of cytoplasmic filamin A was significantly decreased and the expression of nuclear filamin A was increased. This effect was not attenuated by ATPR treatment. *P < .05, **P < .01, ^△^ *P* > .05

To investigate the specific effects of ATPR on the binding of 14‐3‐3ε and filamin A, we analyzed the expression of filamin A in ATPR‐treated gastric cancer cells in the total protein level, cytoplasm, and nuclear fraction, respectively. Subcellular expression analysis showed that filamin A was transferred from the cytoplasm to the nucleus after ATPR treatment and showed a significantly high nucleus to cytoplasm ratio for 14‐3‐3ε and filamin A in the ATPR group, which suggested that ATPR was able to regulate the subcellular localization of filamin A (Figure 5A). According to our previous study, ATPR also regulates the subcellular localization of 14‐3‐3ε and promotes its transfer to the nucleus.^12^ As the binding of 14‐3‐3ε to filamin A decreases after ATPR treatment, 14‐3‐3ε was speculated to promote the transfer of filamin A from the cytoplasm to the nucleus. We transfected SGC‐7901 cells with lentiviral particles and established the 14‐3‐3ε overexpression model. When 14‐3‐3ε was overexpressed, the expression of filamin A was increased. The subcellular distribution of filamin A was changed and increased in the cytoplasm (Figure 5B). When the 14‐3‐3ε overexpression cells were treated with ATPR, the expression of filamin A was decreased and its subcellular distribution was reversed (Figure 5B). The trend of ATPR‐induced G_0_/G_1_ phase arrest was attenuated when 14‐3‐3ε was overexpressed (Figures 4B and 5C). When 14‐3‐3ε was specifically silenced with siRNA transfection, the total amount of cytoplasmic filamin A was significantly decreased and the level of nuclear filamin A was increased. This effect was not attenuated by ATPR treatment (Figure 5D). Our results suggested that 14‐3‐3ε might modulate the subcellular localization of filamin A, whereas ATPR might regulate filamin A by reducing the binding of 14‐3‐3ε to filamin A.

### 3.6 The expression of 14‐3‐3ε and filamin A was increased in gastric cancer tissues

**Figure 6:**
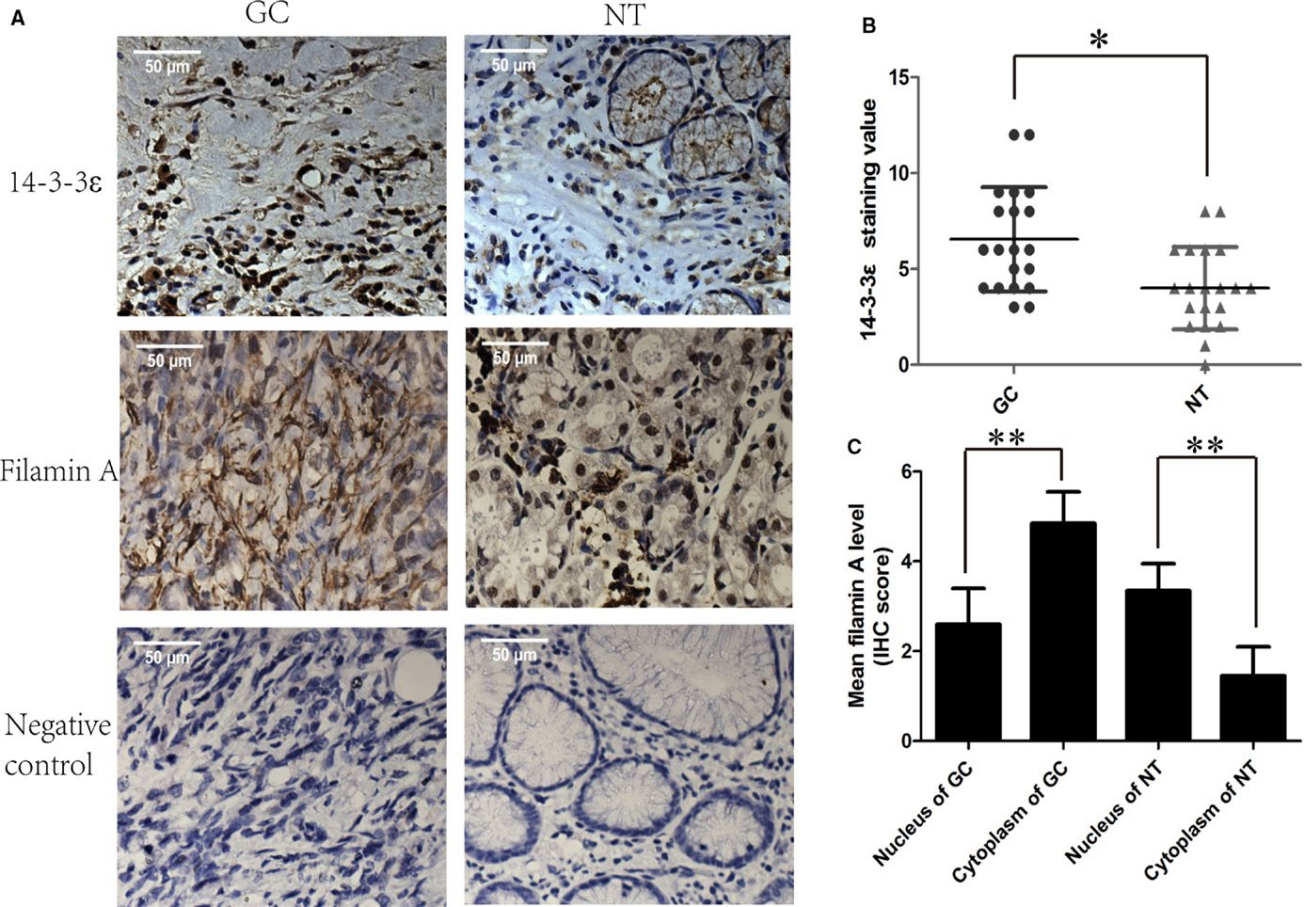
The expression of 14‐3‐3ε and filamin A was increased in gastric cancer tissues. A, Representative immunohistochemical staining of 14‐3‐3ε and filamin A in 20 paired gastric cancer tissues (GC) and adjacent nontumorous gastric tissues (NT) (400×). For negative controls, the primary antibody was substituted with PBS. B, Statistical analysis of the 14‐3‐3ε staining value. C, Filamin A was observed in NT tissue within the nucleus with weak staining in the cytoplasm compared with GC tissue. *P < .05, **P < .01

The expression of 14‐3‐3ε protein was analyzed by immunohistochemical staining in 20 tumor tissues paired with the adjacent normal tissues (Figure 6A). The results were scored using the semiquantitative integration method. The expression levels of 14‐3‐3ε in each patient were converted to staining values for statistical analysis. The results showed that the expression level of 14‐3‐3ε in gastric cancer tissues was significantly higher than that in the matched tissues (Figure 6B). We also examined the expression of filamin A in these tissues (Figure 6A), and we found its level higher in gastric cancer tissues compared with that of the adjacent tissues. In addition, we found that filamin A was mainly distributed in the nucleus in the adjacent normal tissues. However, there was no such trend in the gastric cancer tissues (Figure 6B). Collectively, these data suggested that the expression of 14‐3‐3ε and filamin A were aberrantly increased in GC tissues.

## 4 DISCUSSION

Regulation of protein function requires specific protein‐protein interactions in many ways to achieve precise biochemical and cellular functions. The 14‐3‐3 protein family is a class of molecular chaperone proteins which interact with a wide range of proteins, including transcription factors, biosynthetic enzymes, cytoskeletal proteins, signaling molecules, apoptosis factors, and tumor suppressors, thus playing key roles in many cellular processes.^15^ Therefore, it is important to study the molecular mechanisms of the 14‐3‐3 protein‐dependent regulation. As 14‐3‐3 isoforms are well recognized for their roles in apoptosis, cell cycle regulation, and proliferation in healthy cells, aberrant 14‐3‐3 expression has unsurprisingly emerged as instrumental in the development of many cancers and in their prognosis. The 14‐3‐3ε is the most conservative member of the 14‐3‐3 family. It has been shown to upregulate and to function as an oncogene in many tumors. Yan et al^16^ introduced 14‐3‐3ε and Raf‐1 kinase inhibitor (RKIP) into SGC‐7901 cells using molecular cloning techniques. Their results showed that 14‐3‐3ε increased the activity of tumor cells while RKIP did the opposite. 14‐3‐3ε activated the ERK/MAKP signaling pathway and RKIP inactivated it in this process. 14‐3‐3ε and RKIP are colocated in the cell and interacted with each other, which might help to understand the role of 14‐3‐3ε in the pathogenesis of gastric cancer.

Researchers have used a variety of technical means to achieve a comprehensive understanding of the 14‐3‐3‐binding protein. Ye et al^17^ applied a biotin tagging coupled with an amino acid‐coded mass tagging approach to discover 246 new interactors of 14‐3‐3ε in hepatocarcinoma cells, which would shed light on the understanding of the undefined isomer‐specific functions of 14‐3‐3ε. In our present study, the targeting proteomics method was used to analyze the 14‐3‐3ε‐binding proteins in SGC‐7901 cells. Using bioinformatics, the selected 352 14‐3‐3ε‐binding proteins were annotated with GO terms using PANTHER. The biological processes, protein classes, and pathways involved were obtained, and this information was helpful for us to illustrate the mechanism of ATPR. Pathway analyses revealed that 14‐3‐3ε and its binding proteins were mainly involved in EGF receptor signaling pathway and many other signaling pathways (Figure 2C). This enriches the understanding that 14‐3‐3 is mainly involved in cell cycle and apoptosis signaling, as indicated in some reports.^6^ Furthermore, 25 cell cycle‐related proteins and 14 differentiation‐related proteins were found to be binding with 14‐3‐3ε. Among them, filamin A was involved in both the cell cycle and differentiation process, which drew a lot of our attention.

Filamin A is a well‐known actin cross‐linker protein. It involves multiple cell functions, such as cell signaling, cell migration, and adhesion.^18^ Recently, its role in cancer progression was revised to include additional functions different from the previous reports. Initially, disclosed as a cancer protein, filamin A played a dual role in cancer. When confined to the cytoplasm, it has a tumor‐promoting effect by interacting with signaling molecules. If filamin A undergoes proteolysis, the C‐terminal fragment is localized to the nucleus and acts to inhibit tumor growth. Although it has been localized in the nucleus, it may play a role in suppressing tumor growth and in decreasing the invasiveness of cancer by interacting with transcription factors.^19^ Thus, the induction of filamin A translocating from the cytoplasm to the nucleus may be a promising strategy for cancer treatment. Benjamin A Mooso et al^20^ found that, induction of the nuclear localization of filamin A, promoted apoptosis in prostate cancer cells and inhibited the proliferation of transplanted tumors in vitro. In this study, we found that ATPR was able to regulate the subcellular localization of filamin A by promoting its transfer to the nucleus where it regulated the progression of the cell cycle. These regulatory effects were achieved because ATPR reduced the binding of 14‐3‐3ε to filamin A. This indicates that induction of filamin A nuclear localization may play an important role in cell cycle regulation.

In fact, filamin A was described as potential 14‐3‐3‐binding proteins in HEK293 cells and the activation of T cells recruited 14‐3‐3 proteins to the cell membrane and cytoskeleton to function in different T‐cell processes.^21^, ^22^ However, the impact of the binding between 14‐3‐3ε and filamin A in the cell cycle is not clear. It is well‐known that 14‐3‐3ε proteins participate in the cycle process via PI3K/AKT/FOXO and other signaling pathways, and cell cycle deregulation caused by changes in 14‐3‐3ε expression has been implicated in cancer formation.^23^ One published study showed that the ability of cdc25C to activate mitosis was inhibited in cells by knocking down filamin A expression. In the absence of filamin A, progression through G2 was retarded. Meanwhile, filamin A was identified as an interactor for both cdc25c and 14‐3‐3ε.^24^ As shown in the pathway analysis in Figure 2C, the EGF receptor signaling pathway is one of the major pathways and many binding proteins such as filamin A was involved. Filamin A was reported to participate in the EGF receptor signaling pathway, which regulates the migration and growth. EGF‐induced phosphorylation of EGFR and activation of the Raf‐MEK‐ERK cascade was negatively affected by the silencing of filamin A both in vitro and in vivo.^25^ Rajesh K Nallapalli^26^ suggested that in addition to its classically known cytoskeletal function, filamin A also plays an important role in the activation of ERK and AKT signaling pathways during K‐RAS‐induced transformation. Whether or not filamin A is involved in the EGF receptor signaling pathway in ATPR‐induced G_0_/G_1_ phase arrest is waiting for further study. Furthermore, other 14‐3‐3ε‐binding proteins identified in our proteomics experiment need to be investigated, and the colocalization of the two target proteins in nuclear fraction should be analyzed so as to support our present results.

In conclusion, our study analyzed the biological processes, protein classes, and molecular functions of 14‐3‐3ε‐binding proteins in gastric cancer cells before and after ATPR treatment. We found that the binding of 14‐3‐3ε and filamin A was decreased after ATPR treatment. ATPR can regulate the subcellular localization of filamin A by reducing the binding of 14‐3‐3ε and filamin A, which may be the mechanism of ATPR‐induced cell cycle arrest. Therefore, targeting 14‐3‐3ε and its binding proteins could be a promising strategy for cancer treatment.

## CONFLICT OF INTEREST

The authors have no conflict of interest.
